# A Comprehensive *Ex Vivo* Functional Analysis of Human NKT Cells Reveals Production of MIP1-α and MIP1-β, a Lack of IL-17, and a Th1-Bias in Males

**DOI:** 10.1371/journal.pone.0015412

**Published:** 2010-11-03

**Authors:** Jennifer E. Snyder-Cappione, Camilla Tincati, Ijeoma G. Eccles-James, Amedeo J. Cappione, Lishomwa C. Ndhlovu, Laura L. Koth, Douglas F. Nixon

**Affiliations:** 1 Department of Medicine, University of California San Francisco, San Francisco, California, United States of America; 2 EMD Millipore, Danvers, Massachusetts, United States of America; New York University, United States of America

## Abstract

NKT cells contribute to the modulation of immune responses and are believed to be important in the pathogenesis of autoimmune and infectious diseases, as well as cancer. Variations in the composite NKT cytokine response may determine individual disease susceptibility or severity. Due to low frequencies in peripheral blood, knowledge of the breadth of *ex vivo* human NKT cell functions has been limited. To bridge this gap, we studied highly purified NKT cells from PBMC of healthy donors and assessed the production of 27 effector functions using sensitive Elispot and multiplex bead assays. We found the *ex vivo* human NKT cell response is predominantly comprised of the chemokines MIP1-α, and MIP1-β as well as the Th1 cytokines IFN-γ and TNF-α. Although lower in magnitude, there was also significant production of IL-2, IL-4, and perforin after mitogen stimulation. Surprisingly, little/no IL-5, IL-6, IL-10, or IL-13 was detected, and no subjects' NKT cells produced IL-17. Comparison of the NKT functional profiles between age-matched male and female subjects revealed similar IL-4 responses, but higher frequencies of cells producing IFN-γ and MIP1-α, from males. There were no gender differences in the circulating NKT subset distribution. These findings implicate chemokines as a major mechanism by which NKT cells control responses in humans. In addition, the panoply of Th2 and Th17 cytokine secretion by NKT cells from healthy donors may not be as pronounced as previously believed. NKT cells may therefore contribute to the gender bias found in many diseases.

## Introduction

NKT cells are a rare subset of T lymphocytes with functional characteristics spanning both the innate and adaptive arms of an immune response. NKT cells recognize glycolipid antigens presented via the non-classical MHC CD1d, and can also be activated via Toll like receptor engagement[1]. Populations of NKT cells secrete Th1 and Th2 cytokines[2]–[10]; mouse NKT cells also produce IL-17[11]–[14], a cytokine implicated in the pathogenesis of many autoimmune diseases[15]. While human CD56+TCRβ^+^ cells secrete IL-17[13], whether human NKT cells secrete this Th17 cytokine *ex vivo* is unclear. Human NKT clones have been shown to down-regulate IL-17 production from memory CD4+ T cells[16]. NKT cells contribute to responses against foreign, self, and tumor antigens, and are thought to play a pivotal role in disease progression, including cancer metastasis, where they are now targeted in clinical trials[17]. Paradoxically, NKT cells combat disease progression in certain cases but are associated with poor outcomes in others[2], [18]–[32]. These seemingly conflicting data reflect nuances of NKT cell biology that are currently unknown.

In order to establish how NKT cells modulate immune responses, it is first necessary to determine the breadth and relative magnitude of effector functions exerted *ex vivo* by this T cell population in humans. However, due to the inherent technical difficulties in studying these rare populations (usually less than 0.1% of lymphocytes in PBMC)[9], there is sparse data regarding their patterns of effector functions *ex vivo*. Previous *ex vivo* functional profiling studies used multi-dimensional flow cytometry for simultaneous discrimination and assessment of up to ten NKT cell functions, with reported IL-4, IL-5, IL-10, and IL-13 secretion from ∼10–20% of total NKT cells[3], and enrichment of Th2 cytokines (IL-4, IL-13) within the CD4+ subset[3], [8], [9]. Given the paucity of circulating NKT cells, and alteration of CD4 expression after PMA stimulation[33], flow-based cytokine analysis of bulk PBMCs may provide only limited sensitivity and resolving capacity.

Several diseases reportedly mediated by NKT cells are also strongly influenced by gender. NKT cells influence disease course in several autoimmune disorders[34] as well as tumor progression[35], two types of diseases that also present strong gender biases. For example, systemic lupus erythematosus (SLE), myasthenia gravis (MG), and rheumatoid arthritis (RA) are more common in women than men[36]. Gender specific differences in gene profiles of tumor samples from lung cancer patients have also been reported[37]. Also, IFN-γ secretion from mouse NKT cells is influenced by estradiol[38]. Whether there are sex-related differences in human NKT function is currently unknown.

In this study, we sought to define the scope and magnitude of the *ex vivo* functional capacity of human NKT cells and further assess for gender-specific differences. To answer these questions, we purified NKT cells from freshly isolated PBMC of healthy donors and determined the production of 27 different analytes by sensitive Elispot and Luminex assays. Additionally, eight-color flow cytometry was performed on PBMC from all donors to compare the NKT cell subset distribution between males and females.

## Results

### Study Subjects, NKT gating strategy, and purity of sorted NKT cell populations

**Figure 1:**
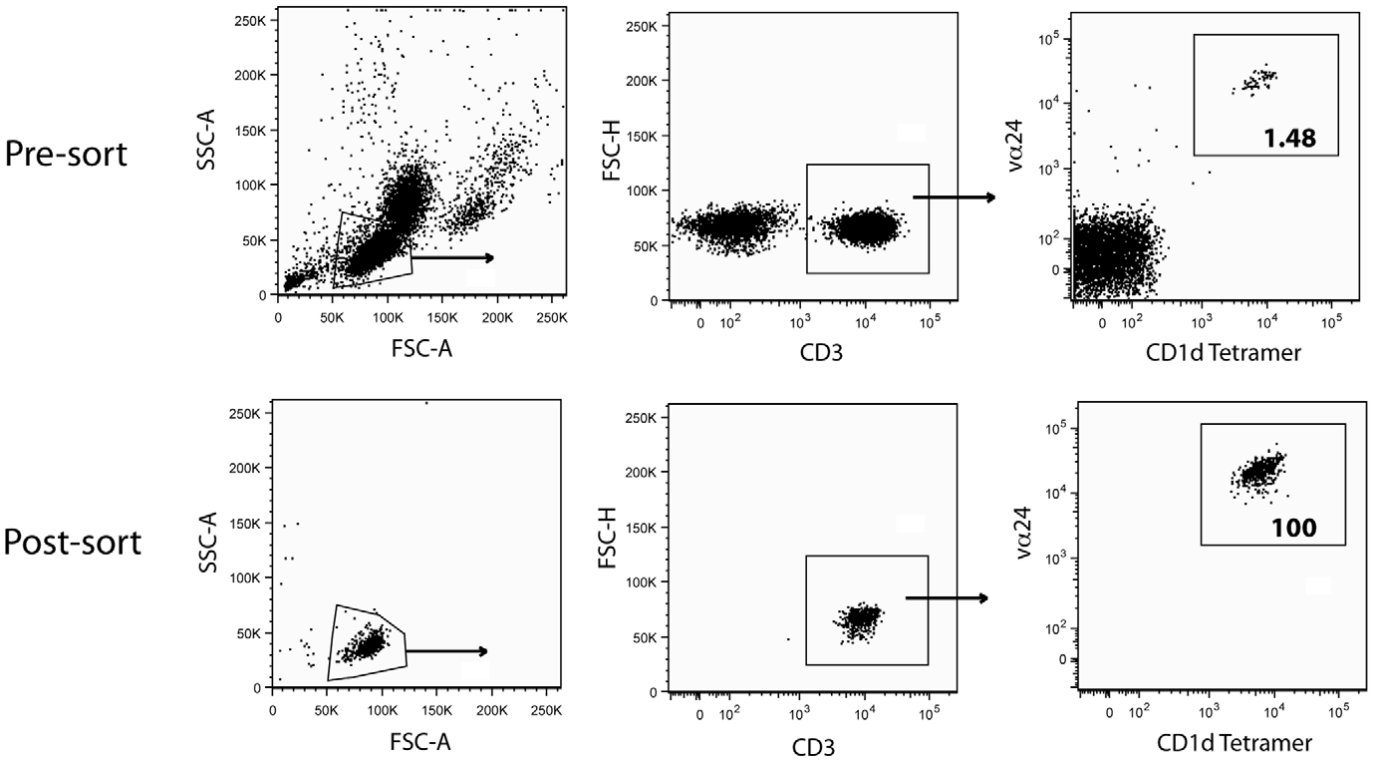
NKT gating strategy and an example of sorting purity. PBMC were first gated on lymphocytes, and NKT cells were visualized as CD3+, vα24+ and CD1d-PBS57 tetramer+. The percentage of NKT cells within the CD3 gate is shown in the far right plots. An example of pre-and post-sort plots from one representative donor are shown. Abbreviations: FSC-A, Forward Scatter Area; SSC-A, Side Scatter Area; FSC-H, Forward Scatter Height.

Due to their low numbers in human peripheral blood, our understanding of the *ex vivo* functional capabilities of NKT cells is limited. To better elucidate the range of cytokine, chemokine, and cytotoxic-associated NKT responses directly *ex vivo*, we purified this unique T cell population using flow cytometric cell sorting. The subjects in our cohort had a wide range of NKT cell frequencies (1.010 to 0.009 per cent of lymphocytes) and the sort purities were high for all subjects, averaging greater than 99 percent (Table 1). We collected approximately 100 ml of fresh blood from 12 healthy donors (six males and six females). We discriminated NKT cells via expression of CD3, vα24, and a CD1d-antigen loaded tetramer to ensure accurate gating[8], [9], [39] (Figure 1).

**Table 1:** Donor information with sorting purity.

Donor	Sex	Age	Ethnicity	NKT frequency (% lymphocytes)	Post-Sort Purity
1	M	29	Asian	1.010	100%
2	F	22	Caucasian	0.660	99.9%
3	M	31	Asian	0.600	99.9%
4	F	24	Caucasian	0.590	99.8%
5	M	32	Caucasian	0.410	100%
6	M	30	Caucasian	0.240	99.8%
7	M	32	Caucasian	0.110	98.5%
8	F	29	Caucasian	0.098	99.5%
9	F	31	Caucasian	0.071	97.5%
10	F	23	Caucasian	0.057	99.4%
11	M	33	Caucasian	0.016	98.1%
12	F	30	Asian	0.009	96.7%

Subjects are listed in descending NKT cell frequency. Sort purities are expressed as percent of CD3+ T cells.

### Single cell resolution of the ex vivo effector functions of human NKT cells via Elispot assays

**Figure 2:**
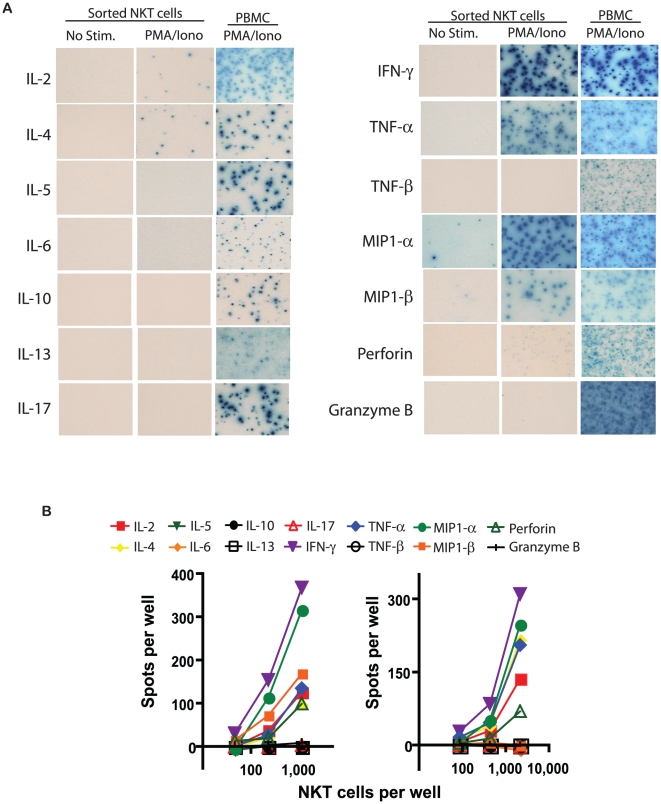
Measurement of the *ex vivo* effector fuctions of purified NKT cells by elispot assays. Determination of the frequencies of NKT cells exerting 14 different effector functions via elispot assays. (A) Representative images of elispot wells with either sorted NKT cells with no stimulation, sorted NKT cells with PMA and Ionomycin, or total PBMC with PMA and Ionomycin (positive controls). (B) NKT cell dilution results from two donors. Each symbol represents one effector function.

Human NKT cells have been reported to make several Th1 and Th2 cytokines, with the relative magnitude of each potentially influencing disease course. To compare the frequencies of NKT cells exerting a broad spectrum of effector functions, we performed 14 different elispot assays, including Th1 cytokines (IL-2, IFN-γ, TNF-α, TNF-β), Th2 cytokines (IL-4, IL-5, IL-6, IL-10, IL-13), the Th17 cytokine IL-17, chemokines (MIP1-α, and MIP1-β), and cytotoxic effector molecules (perforin, granzyme B) (Figure 2A). Serial dilutions of sorted cells were performed for all assays in the majority of subjects (cell number permitting); the resulting linear relationship between input cell number and spot number in the elispot assay confirms each spot accurately represents one cytokine-producing cell (Figure 2B).

### Elispot assays detect NKT cells secreting seven of the 14 functions measured in response to mitogen stimulation

**Figure 3:**
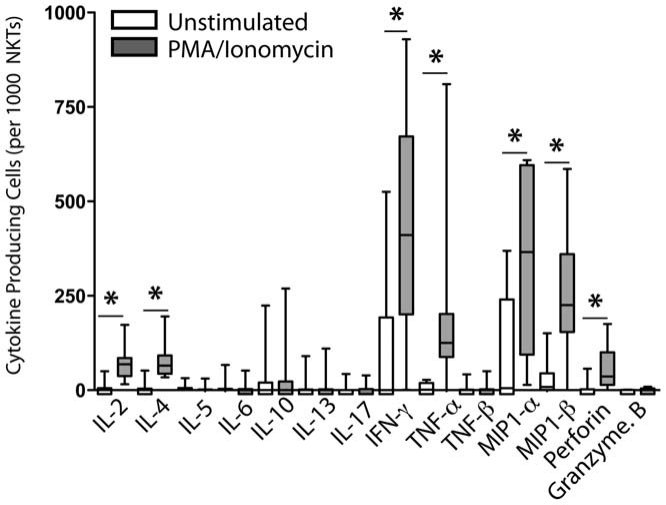
Total frequencies of NKT cells exerting different effector functions *ex vivo* from healthy donors. The data is expressed as box and whisker plots, with the median for all subjects shown as the center line, the box representing the 25–75 percentile, and the lines showing the range of the data. Statistically significant differences between unstimulated (white boxes) and stimulated (grey boxes) wells (p<0.05) are noted with an asterisk. Eight subjects were included in this analysis.

We next determined the total frequencies of NKT cells exerting 14 different functions *ex vivo* for all subjects in the cohort. When comparing the number of spots from unstimulated and stimulated wells, we found significant induction of spots by PMA and Ionomycin for seven of the 14 analytes, including IL-2, IL-4, IFN-γ, TNF-α, MIP1-α, MIP1-β, and Perforin (Figure 3). IL-5, IL-6, IL-10, IL-13, and IL-17-producing NKT cells were not detected following mitogen exposure, but were observed in the positive controls (Figure 3).

### Luminex assays confirmed little/no IL-5, IL-6, IL-10, and IL-13 production and a lack of IL-17 secretion from the NKT cells of healthy donors

**Figure 4:**
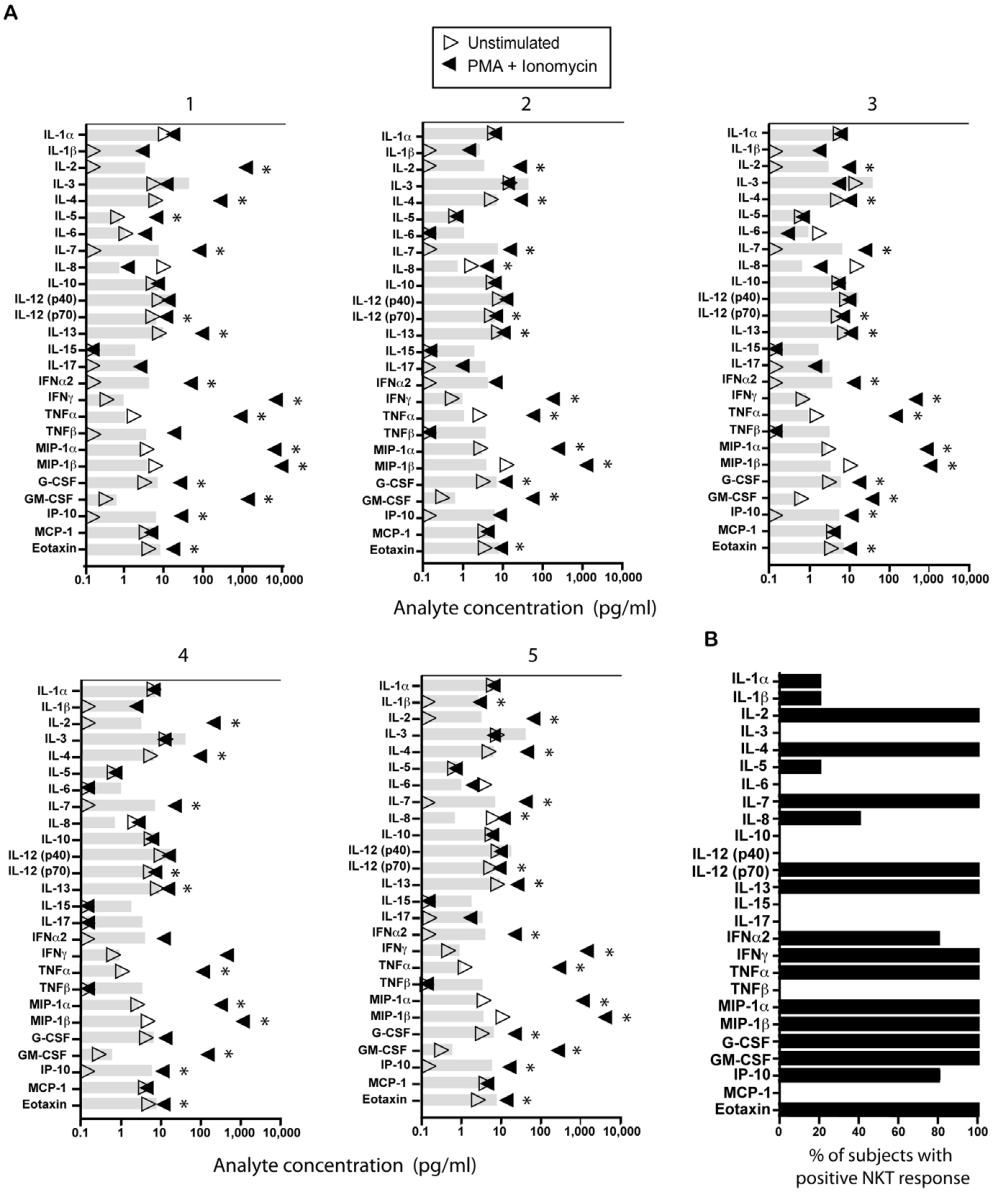
26-plex Luminex assays reveal the *ex vivo* functional bio-signature of human NKT cells. The levels of 26 different analytes were measured from culture supernatants of pure NKT cell populations, either with or without PMA and Ionomycin stimulation. In (A), each plot represents one individual, with the donor number noted at the top. The limits of detection of each analyte are shown in gray bars, and are listed in the materials and methods. A significant level of analyte (as defined in the materials and methods) from either unstimulated and/or stimulated culture supernatants is noted with an asterisk. Symbols on the Y axis represent values to low to be given a numerical value. Note that the scale is different for the donor 1 graph (0.1–11,000). (B) The percentage of the five subjects whose culture supernatants (either unstimulated, stimulated, or both) gave a significant signal for each analyte.

To confirm our elispot findings and expand our analysis of NKT cell functions, we next performed 26-plex Luminex assays on supernatants from one to two day cultures of freshly sorted peripheral blood NKT isolates. As expected, we found significant production of the cytokines detected by our elispot experiments in response to PMA and Ionomycin, (IL-2, IL-4, IFN-γ, TNF-α, MIP1-α, MIP1-β) (Figure 4A) for all five subjects (Figure 4A and 4B). Also, overall, we found extremely low levels of IL-5, IL-6, IL-10, or IL-13 in the subject supernatants. One exception was found for subject 1, where IL-13 secretion was more than 10 fold over the assay's limit of detection (Figure 4A).

Measurement of IL-17 via Luminex assays revealed this hallmark Th17 cytokine was not present in culture supernatants from any of the donors (Figure 4A and 4B). These data, together with our elispot results, conclusively show a lack of IL-17 production *ex vivo* from the NKT cells of all donors in our cohort.

### NKT functional profiles of age-matched subjects reveal a significant Th1 bias in male donors

**Figure 5:**
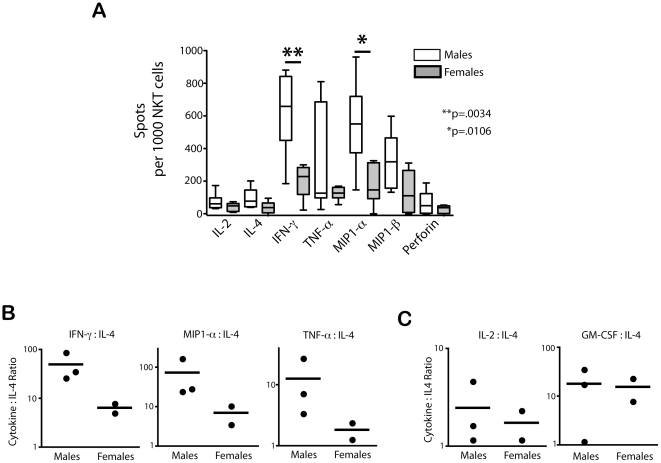
Th1 bias of NKT cells from male donors. The ex vivo effector functions of NKT cells from male and female subject groups were compared. (A) The gender differences (n = 6/group) in the number of cytokine producing cells detected via elispot; statistically significant differences between groups are noted. White and grey boxes denote the male and female groups, respectively. Data is shown as box and whisker plots, with the center line noting the median, the boxes noting the 25–75 percentile, and the lines representing the range for each group. The ratio of Th1 cytokines (B) and non-Th1 cytokines (C) to IL-4 from pure NKT cell populations measured by the luminex assay. In (B) and (C), each circle represents one donor, with the line noting the mean for each group.

As there are noted sex differences in susceptibility to many diseases whose progression may be influenced by NKT cells, we performed a gender-based comparative analysis of NKT effector function profiles. The frequencies of NKT cells secreting IL-2 and IL-4 after mitogen stimulation were similar between male and female subject groups (Figure 5A). However, males had significantly greater numbers of circulating NKT cells that produce the Th1 cytokines IFN-γ and MIP1-α in response to PMA and Ionomycin than their female counterparts (Figure 5A). The number of TNF-α–secretingNKT cells varied considerably amongst male subjects, but was comparatively lower amongst all female subjects, with less than 17% of NKT cells secreting this major Th1 cytokine after stimulation (Figure 5A). As our elispot assays provide information on the number of cells making a cytokine, but not the quantity cytokine made, our next question was whether male-derived NKT cells also secrete a higher ratio of Th1:Th2 cytokines. To address this, we compared the cytokine concentration values derived from our Luminex assays. We found higher mean ratios for Th1 cytokines IFN-γ, MIP1-α, and TNF-α to IL-4 amongst male subjects as compared to the female group, (49∶6, 72∶7, and 13∶2, respectively) (Figure 5B). In contrast, we found no apparent gender difference when comparing the ratio of IL-4 to the cytokines IL-2 or GM-CSF, neither of which is exclusively secreted by Th1 cells (Figure 5C). Taken together, these data demonstrate a Th1 bias of *ex vivo* NKT cell functions in males.

### No differences in the subset distribution of circulating NKT cells between men and women

**Figure 6:**
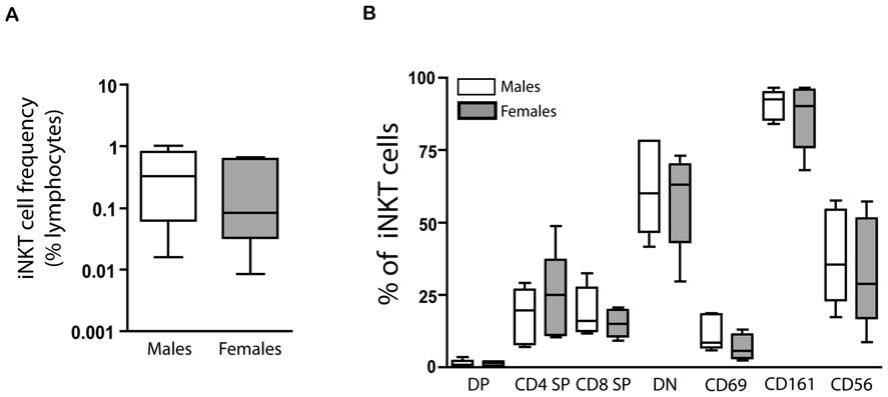
Similar distribution of NKT cell subsets between sexes. Eight-color flow cytometry was performed on PBMC samples from the same blood draw as the sorted NKT cell populations to compare (A) the NKT total cell frequencies as a per cent of lymphocytes and (B) the CD4+ CD8+ (Double Positive, or DP), CD4+CD8− cells (CD4 SP), CD4− CD8+ (CD8 SP), CD69+; CD161+, and CD56+ NKT cell subsets between male (white boxes) and female (grey boxes) subject groups. No significant differences were found. Data is shown as box and whisker plots, with the center line noting the median, the boxes noting the 25–75 percentile, and the lines representing the range for each group.

Subsets of human NKT cells have been reported to exert different functional patterns, with CD4− CD8− (DN) cells secreting only Th1 cytokines, while the CD4+ CD8− (SP) fraction produces both Th1 and Th2 cytokines [3], [8], [9]. As our data found gender-related differences in the effector function profiles of the circulating NKT cell compartment (Figure 5A–C), we next sought to determine whether this functional divergence was due to differences in circulating NKT subset distributions. Using flow cytometry, we measured both the total NKT frequencies and the percentage of CD4, CD8, CD69, CD56, and CD161 NKT subsets within PBMC from the same blood draw as our sorted samples from all subjects. We found no significant differences for either total NKT frequencies (Figure 6A) or the percentages of CD4+ CD8+ (DP), CD4+ CD8− (SP), CD4− CD8+ (SP), CD4− CD8− (DN), CD69+, CD161+, or CD56+ subsets between the male and female subject groups in our study (Figure 6B).

## Discussion

This study measured the *ex vivo* functional profiles of purified human NKT cells and demonstrated that this T cell population exerts a lower Th2 response, both in magnitude and diversity, than previously reported. Also, IL-17 was not detected from any NKT cell populations in this study. In addition, we found that the chemokines MIP1-α and MIP1-β were two of the most predominant factors secreted from the NKT cells of all subjects in our cohort. These experiments also revealed that male NKT cells are more Th1-biased than their female counterparts.

Our data revealed the significant production of some factors not previously associated with NKT cell effector function profiles. For example, the NKT cell populations from the vast majority of subjects in our cohort secreted the chemokines MIP1-α and MIP1-β(Figures 4, 5), at levels similar to IFN-γ (Figures 3, 4). MIP1-α and MIP1-β are potent inflammatory agents that attract both monocyte and lymphocyte subsets. NKT cells often influence immune responses via stimulation of dendritic cells[40], [41]. Therefore, future studies to determine whether NKT cells control dendritic cell recruitment via these chemokines is of high interest. Other factors, including eotaxin, IP-10, and IL12p70 were found at low but detectable levels in the NKT culture supernatants from most donors in this study. Due to the low amounts measured, these findings may be due to a minute fraction of contaminating (non-NKT) cells; further experiments are necessary to confirm the NKT specificity of these analytes. Further exploration of these factors could lead to a greater understanding of how NKT cells influence disease onset and pathogenesis. Also, the NKT-specific expression of these analytes may be novel biomarkers of disease states and thus provide valuable tools for predictive diagnostics as well as new therapeutic targets.

Our results found little/no IL-5, IL-6, IL-10, and IL-13 secreted by NKT cells from the healthy donors in our study (Figures 2, 3, 4). These results are in contrast with some previous flow cytometric studies[3], [8]. As flow cytometric intracellular staining detects production of cytokines, and elispots measure release of proteins from cells, it is possible that these cytokines are modified post-translationally within NKT cells to inhibit their secretion. Alternatively, performing multiparameter intracellular flow cytometric analysis on these rare cells could result in artifacts within the Th2 cytokine gates, especially when data is generated from low event counts and imprecise NKT gating. Taken together, our Elispot and Luminex bead array data implicate IL-4 as the predominant Th2 cytokine secreted by circulating human NKT cells. We therefore contend that IL-4 should be a major target when considering therapeutic immuno-modulation of NKT cells to control inflammatory disease course.

Another cytokine that was not detected from any NKT cell populations in our study is IL-17 (Figures 2, 3, 4). From these results, we conclude that circulating human NKT cell populations may not include a Th17 differentiated subset, unlike what has been found in mice[11]–[14]. As human NKT cells specifically suppress the Th17 arm of an immune response[16], it is possible there are divergent roles of mouse and human NKT cells in controlling immunity. This is important to note when performing mouse studies of human NKT-related diseases, as the abstraction of animal studies to human clinical trials may be misleading. However, as our studies were performed in healthy donors, it is of interest to determine whether IL-17-producing NKT cells are found in diseased individuals.

We found a significant Th1 bias of the functional NKT profile of male cells as compared to those from female donors. This finding was evident from both assays (Elispot and Luminex assays) revealing differences in both the relative frequencies of Th1 and Th2 producing NKT cells and the total amount of cytokine from each lineage secreted into the culture supernatant from mitogen stimulated short-term cultures. These data may help explain the gender-based propensities associated with certain diseases. Also, we were surprised to find similar distributions of several NKT subsets in the PBMC of our male and female groups. We therefore propose that *in vivo*, the NKT cell effector function profile may be influenced by external factors in the local microenvironment, such as hormones or other factors. Future studies to determine whether the functions of male and female NKT cells gain similarity after long term culture *in vitro* is of interest, and may reveal novel ways to modify NKT cell responses to direct favorable immune responses in the host.

In summary, by using FACS-Aria cell sorting to purify freshly isolated NKT cells from human donors and then measuring the capacity of this unique T cell population to exert 27 different effector functions *ex vivo*, we report several new observations about this unique T cell subset that provide a more comprehensive understanding of their functional profiles. Also, these experiments revealed a novel functional Th1-bias of male NKT cells. Future work to measure alteration of this functional profile in disease states could provide novel insight into how NKT cell modulate immune responses and influence disease pathogenesis.

## Materials and Methods

### Subjects

Healthy subjects were recruited by the Division of Experimental Medicine at the University of California, San Francisco (San Francisco, CA, USA). All subjects were at least 21 years of age at the time of sample collection (Table 1). All samples were obtained according to protocols approved by the Research Subjects Review Board at UCSF. Written informed consent was obtained from all subjects.

### Human lymphocyte preparation

Blood was drawn into EDTA tubes and PBMC were isolated within 16 hours of collection using ficoll-hypaque, washed twice with PBS, and resuspended in FACS buffer (PBS with 0.5% BSA and 2 mM EDTA) for staining.

### Fluorescent antibodies and tetramers

For the sorting and determination of circulating NKT subsets, the following reagents were used: PE-labeled CD1d-tetramer loaded with PBS57 from the NIH Tetramer Facility (located at Emory University, Atlanta, GA, USA). Other reagents used in the phenotypic analysis of PBMC include: anti-CD3 ECD and anti-vα24 biotin (Beckman Coulter, Fullerton, CA, USA) and Streptavidin-Qdot 655 (Invitrogen, Eugene, OR, USA); anti-CD56 PE-Cy7, anti-CD161 APC, and anti-CD4 Alexa 700 (BD Biosciences, San Jose, CA, USA); and anti-CD8 Qdot 605 (UCSF), and Amine Aqua was used for live/dead discrimination (Invitrogen, Eugene, OR, USA).

### NKT Cell Sorting

Freshly isolated PBMC were incubated with CD1d-tetramer-PBS57, anti-CD3, and anti-vα24 antibodies for 30 minutes at 4°C. Cells were then washed twice with FACS buffer (PBS with 0.5% BSA and 2 mM EDTA), and Streptavidin-Qdot 655 was added for 20–30 minutes at 4°C. Cells were washed twice with FACS buffer, and sorted on a FACS-Aria flow cytometer (BD Biosciences, San Jose, CA, USA). All cells were stained at a concentration of 1×10^7^ PBMC per 100 µl of antibody mixtures in v-bottom 96 well plates. For all sorts, doublets were excluded from the sorted population via gating discrimination using FSC-A and FSC-H. Purities shown reflect the percentage of CD3+ cells in the population. The vast majority of the few non-NKT events were found outside the FSC-SSC gate, in the area of debris (Figure 1).

### Elispot antibodies

The following reagents were used for plate coating: anti-IL-2 (1∶60 dilution, R&D systems, Cat. #SEL202, Minneapolis, MN USA), anti-IL-10 (1∶60 dilution, R&D systems, Cat. #SEL217B, Minneapolis, MN USA), anti-IL-13 (1∶60 dilution, R&D systems, Cat. #SEL213, Minneapolis, MN USA), anti-IL-4 (15 µg/mL, Mabtech, Cat.#3410-3-250, Nacka Strand, Sweden), anti-IL-5 (5–10 µg/mL, BD Biosciences, Cat.# 554393, San Jose, CA, USA), anti-IL-6 (5 µg/mL, Mabtech, Cat. #3460-3-250, Nacka Strand, Sweden), anti-IL-17 (5 µg/mL, ebiosciences, Cat.# 14-7178-85, San Diego, CA, USA), anti-IFN-γ (10 µg/mL, Mabtech, Cat.# 3420-3-1000, Nacka Strand, Sweden), anti-TNFα (5 µg/mL, Mabtech, Cat.# 3510-3-1000, Nacka Strand, Sweden), anti-TNF-β (10 µg/mL, R&D Systems, Cat.# MAB621, Minneapolis, MN, USA), anti-MIP1-α (10 µg/mL R&D systems, Cat.# MAB270, Minneapolis, MN, USA), anti-MIP1β (5 µg/mL, Mabtech, Cat.# 3495-3-250, Nacka Strand, Sweden, anti-perforin (5 µg/mL, Mabtech, Cat.# 3465-3-1000 Nacka Strand, Sweden), anti-granzyme B (15 µg/ml, Mabtech, Cat.# 3485-3-1000, Nacka Strand, Sweden). The following biotinylated antibodies were used: anti-IL-2 (1∶60 dilution, R&D systems, Cat. # SEL202, Minneapolis, MN USA), anti-IL-10 (1∶60 dilution, R&D systems, Cat. # SEL217B, Minneapolis, MN USA), anti-IL-13 (1∶60 dilution, R&D systems, Cat. # SEL213, Minneapolis, MN USA) anti-IL-4 (2 µg/mL, Mabtech, Cat.# 3410-6-250, Nacka Strand, Sweden), anti- IL-5 (2 µg/mL, BD Biosciences, Cat.# 554491, San Jose, CA, USA), anti-IL-6 (1 µg/mL, Mabtech, Cat. #3460-6-250, Nacka Strand, Sweden), anti-IL-17 (1 µg/mL, ebiosciences, Cat.# 88-7876-DT, San Diego, CA, USA), anti-IFN-γ (1 µg/mL, Mabtech, Cat.# 3420-6-250, Nacka Strand, Sweden), anti-TNF-α (2 µg/mL, Mabtech, Cat.# 3510-6-1000, Nacka Strand, Sweden), anti-TNF-β (1 µg/mL, R&D Systems, Cat.# BAF211, Minneapolis, MN USA), anti-Mip1α (2 µg/mL, R&D systems, Cat.# BAF270 Minneapolis, MN USA), anti-MIP1-β (1 µg/mL, Mabtech, Cat.# 3495-6-250, Nacka Strand, Sweden), anti-perforin (1 µg/mL, Mabtech, Cat.# 3465-6-1000, Nacka Strand, Sweden), anti-granzyme B (1 µg/ml, Mabtech, Cat.# 3485-6-1000 Nacka Strand, Sweden).

### Elispot assays

Elispot plates (#MAIPN4550; Millipore, Danvers, MA, USA) were coated with primary antibodies for a minimum of one hour at room temperature, and washed twice with PBS before cells were added. The pure NKT cell populations were added in 200 µl of complete media; the range of the top dilutions for all donors was 3,333 to 118 cells/well. Cells were added to wells with no stimulation (eight of the 12 donors) and/or PMA (50 ng/mL) and Ionomycin (500 ng/mL) (12 of the 12 donors). Due to low cell yields, four donors' NKT cells were only added to wells with PMA/Ionomycin. Cell number permitting, multiple dilutions were performed for all assays to ensure accurate spot numbers (Figure 2B). The plates were incubated at 37°C, 5% CO_2_ for 15–18 hours. At the end of the culture period, the plates were washed twice with PBS and twice with PBS plus 0.1% Tween 20 (PBST), and secondary antibodies were added in PBS 0.1% Tween 20 1% BSA (PBSTB), 50 µl/well for 45 minutes at room temperature. Plates were then washed again twice with PBST, and the streptavidin-Alkaline Phosphatase (1∶1000 dilution, Jackson Immunoresearch, Cat.# 016-050-084, West Grove, PA, USA) was added in 100 µl PBSTB for 45 minutes. Plates were again washed twice with PBST, the plastic backing was removed, and then the plates were immersed in PBST for one hour. Next, substrate mix was added (Vector Laboratories, Cat. #SK5300, Burlingame, CA, USA). When blue spots were clearly visible, the plates were washed with tap water. When plates were dry, spots were counted using an automated elispot reader.

### NKT cell cultures for supernatant collection

NKT cell populations from five donors (11,000–50,000 cells per well) were cultured for one-two days either with or without mitogen stimulation. At the end of the culture period, plates were spun and supernatants were carefully collected and frozen at −20°C until thawed for Luminex analysis.

### Luminex assays

Relative content and concentration for 26 analytes was determined using cytometric multi-analyte technology. Specifically, 26-plex human cytokine/chemokine bead array kits (MILLIPLEX™ MAP, Millipore Corporation, USA) were used in conjunction with a Luminex 100 platform following manufacturer's instructions. Briefly, 25 µl sample (or cytokine standard mix) was mixed with 25 µl pooled capture beads and incubated for one hour at RT under gentle agitation. Samples were washed by centrifugation and then mixed with biotinylated detection antibody. After a one-hour incubation, streptavidin-PE detection reagent was added and the assay was carried out for an additional 30 minutes. Samples were washed, resuspended in storage buffer, and placed at 4°C until analysis. For each analyte, a minimum of 50 bead events was collected. All samples were run in duplicate. Raw data was analyzed using MILLIPLEX Analyst software. Standard curves were generated (in duplicate) from lyophilized standard provided with each kit. The concentration for each analyte in cell supernatants was determined by interpolation from their corresponding standard curve. The limits of detection for each analyte are the lowest detectable point on the standard curve + two standard deviations. For each analyte, the limits of detection (in pg/ml) were: eotaxin: 7.8, G-CSF: 7.7, GM-CSF: 3.5, IFN-α2: 4.0, IFN-γ: 3.3, IL-1α: 8.6, IL-1β: 2.5, IL-2: 3.2, IL-3: 41.6, IL-4: 7.8, IL-5: 3.2, IL-6: 5.1, IL-7: 7.1, IL-8: 3.7, IL-10: 8.0, IL-12(p40): 18.0, IL-12(p70): 6.9, IL-13: 10.1, IL-15: 1.8, IL-17: 3.4, IP-10: 6.2, MCP-1: 5.3, MIP1-α: 3.4, MIP1-β: 7.3, TNF-α: 3.6, and TNF-β: 3.4. Significant responses (defined with an asterisk in figure 4A and graphed in figure 4B) were defined as average values greater than the limit of detection with and less than 20% CV value between duplicates. Undetectable levels from culture supernatants were given an arbitrary value of zero and are located on the Y axis in Figure 4A.

### Flow cytometry to determine circulating NKT subset distribution

PBMCs were incubated with CD1d-tetramer-PBS57, anti-CD3, anti-CD56, anti-CD161, anti-CD4, Amine Aqua, anti-CD8, and anti-vα24 antibodies for 30 minutes at 4°C. Cells were then washed twice with FACS buffer, and streptavidin-Qdot 655 was added for 20–30 minutes at 4°C. Cells were washed twice with FACS buffer, resuspended in 2% paraformaldehyde, and run on a LSR-II Flow Cytometer (BD Biosciences, San Jose, CA, USA). All cells were stained at a concentration of 1×10^7^ PBMC per 100 µl of antibody mixtures in v-bottom 96 well plates. The data were analyzed with FlowJo software (version 8.5.2, Tree Star, Ashland, OR, USA). Gating and analysis of all data was performed without knowledge of the subjects' characteristics, including gender.

### Statistical analysis

A two-tailed T test was used to determine significant differences between groups in Figure 3, Figure 5A, Figure 6A, and Figure 6B. A p value of <0.05 was considered statistically significant.
